# Computation in Complex Networks

**DOI:** 10.3390/e23020192

**Published:** 2021-02-05

**Authors:** Clara Pizzuti, Annalisa Socievole

**Affiliations:** National Research Council of Italy (CNR), Institute for High Performance Computing and Networking (ICAR), Via Pietro Bucci, 8-9C, 87036 Rende, Italy

The Special Issue on “Computation in Complex Networks” focused on gathering highly original papers in the field of current complex network research. Due to their ability to model a wide variety of daily-life systems—including the Internet, communication, chemical, neural, social, political and financial networks—complex network systems and their behavior need to be deeply understood. As such, the focus of this Special Issue has been highlighting and promoting current interdisciplinary contributions on the various fields of complex networks, thus providing a collection of high-quality research papers that capture the challenges recently posed by these networks. We selected 20 manuscripts, which are described below.

In the paper “Active Learning for Node Classification: An Evaluation” by Madhawa and Murata [1], the active learning framework was used as a method to make node classification on attributed graphs by representing data instances as nodes of the graph. The authors performed an empirical evaluation of different state-of-the-art active learning algorithms proposed for graph neural networks, as well as other data types, such as images and text, on several real-world attributed graphs. The results showed that active learning algorithms designed for other data types do not perform well on graph-structured data, highlighting the importance of complementing uncertainty-based active learning models with an exploration term.

In the paper “Spreading Control in Two-Layer Multiplex Networks” by Jaquez et al. [2], the problem of controlling an SIS (Susceptible-Infected-Susceptible) epidemic spreading over a network with two layers was addressed. The stabilization of the extinction state for the nonlinear discrete-time model was obtained by properly tuning system parameters, such as intralayer and interlayer transmission rates, for a limited number of nodes characterized by a parametric threshold condition. The sufficient conditions for the choice of the subset of nodes and the parameters to be controlled were established through a rigorous mathematical analysis guaranteeing the exponential stability of the extinction state globally, with respect to the set of all possible probability states.

In the paper “Investigating the Influence of Inverse Preferential Attachment on Network Development” by Siew and Vitevitch [3], the growth mechanism of phonological language networks, in terms of the acquisition of new words that are phonologically similar to existing ones, was explored. Specifically, the authors analyzed the network structure and the degree distributions of networks synthetically generated through preferential attachment, an inverse variant of the classical version where new nodes are connected to existing nodes with fewer edges, or combinations of both network growth mechanisms. The simulation results showed that preferential attachment—followed by inverse preferential attachment—in the network growth resulted in densely connected network structures.

In the paper “Classification of Literary Works: Fractality and Complexity of the Narrative, Essay, and Research Article” by Ramirez-Arellano [4], the problem of the classification of literary works was tackled. This research analyzed the node degree, betweenness, shortest path length, clustering coefficient, nearest neighborhoods’ degree, fractal dimension, complexity, area under box-covering, and area under robustness curve of the complex networks. The literary works of Mexican writers were analyzed, with the aim of classifying them according to their genre. The results of this analysis classified 87% of the full word co-occurrence networks as a fractal.

In the paper “Detecting Overlapping Communities in Modularity Optimization by Reweighting Vertices” by Tsung et al. [5], the community detection problem was considered, specifically focusing on overlapping community sets of nodes. By first introducing a node weight allocation problem to formulate the overlapping property, the authors proposed a genetic algorithm, exploiting an extension of the modularity function for solving the node weight allocation problem and detecting the overlapping communities. Moreover, three refinement strategies for improving the quality of results were added. On both real-world and synthetic networks, the proposed algorithm was able to better detect nontrivial overlapping nodes, compared to other contestant algorithms.

In the paper “Modelling and Recognition of Protein Contact Networks by Multiple Kernel Learning and Dissimilarity Representations” by Martino et al. [6], the authors focused on predicting the proteins’ functional role, proposing a hybrid classification system based on a linear combination of multiple kernels defined over multiple dissimilarity spaces. Here, the training procedure jointly optimized the kernel weights and the representatives’ selection in the dissimilarity spaces. The classification system was thus characterized by a double knowledge discovery phase in which the analysis of the weights allowed the authors to check which representations were better for solving the classification problem—whereas the pivotal patterns selected as representatives give further insight into the modelled system. Experimental results showed how the proposed classification system was able to reliably analyze the considered protein contact networks.

In the paper “Cross-Domain Recommendation Based on Sentiment Analysis and Latent Feature Mapping” by Wang et al. [7], a cross-domain recommendation algorithm (CDR-SAFM) based on sentiment analysis and latent feature mapping was proposed. This algorithm specifically combined the sentiment information extracted from different domains of users’ ratings. The sentiment is categorized into (1) positive, (2) negative and (3) neutral. Moreover, the latent Dirichlet allocation (LDA) was used to model the users’ semantic orientation to generate the latent sentiment review features. Finally, by applying multilayer perceptron (MLP), the CDR-SAFM was able to obtain the cross-domain nonlinear mapping function to transfer the users’ sentiment review features. Tested on the Amazon dataset, the proposed recommendation algorithm outperformed other existing recommendation algorithms in the considered cross-domain scenario.

In the paper “Complex Contagion Features without Social Reinforcement in a Model of Social Information Flow” by Pond et al. [8], the problem of information spreading over social networks through a complex contagion model was considered. Focusing on the quoter model (a model of the social flow of written information copying or “quoting” short subsequences of text from neighbors), the authors showed how this model has features of complex contagion, including the weakness of long ties and the high network density that limits information flow rather than boosting it, despite lacking an explicit mechanism of social reinforcement that distinguishes complex contagion from epidemic spread.

In the paper “Optimizing Variational Graph Autoencoder for Community Detection with Dual Optimization” by Choong et al. [9], variational graph autoencoders for community detection were considered. The research underlined how variational autoencoder (VAE)-based approaches suffer from a deviation increase from the primary objective when minimizing loss using the stochastic gradient descent, resulting in suboptimal community structure. To smooth this effect, a dual optimization procedure was proposed to guide the optimization process toward better communities. The results of the experiments showed that the proposed community detection algorithm outperformed its predecessor.

In the paper “Properties of the Vascular Networks in Malignant Tumors” by Chimal-Eguìa et al. [10], both synthetic and real angiogenic vascular networks of patients with Hepato-Cellular Carcinoma (HCC), extracted from digital tomographies, were analyzed. From the measurements of network properties, such as average path length, clustering coefficient, degree of distribution and fractal dimension, the authors showed that there is a well-connected network (high clustering coefficient), different from previous related works. The network exhibited efficient communication. This was also reflected by the small average path length.

In the paper “Complex Network Construction of Univariate Chaotic Time Series Based on Maximum Mean Discrepancy” by Sun [11], the focus was on the analysis of chaotic time series; more specifically, on how measuring the similarity between time series affected construction of the corresponding network. Here, a method that first transforms univariate time series into high-dimensional phase space, then exploits a Gaussian mixture model (GMM) to represent time series, and finally introduces maximum mean discrepancy (MMD) to measure the similarity between GMMs was proposed. The introduced MMD was validated using the Lorenz system, showing that the similarity between GMMs can be measured more effectively.

In the paper “Analyzing Uncertainty in Complex Socio-Ecological Networks” by Maldonado et al. [12], the aim was to assess the impact of using the Bayesian network structure for modeling complex socio-ecological networks, whose behavior is often uncertain. The conducted analysis was two-fold. The first experiment assessed the impact of the Bayesian network structure on the entropy of the model. The second compared the entropy of the posterior distribution of the class variable obtained from the different structures. For the experiments, three types of Bayesian networks are analyzed: naive Bayes (NB), tree augmented networks (TAN) and networks with unrestricted structure (GSS). The results showed that GSS consistently outperformed both NB and TAN when evaluating the uncertainty of the entire model, while NB and TAN resulted in lower entropy values of the posterior distribution of the class variable, making them suitable for prediction tasks.

In the paper “Multi-Type Node Detection in Network Communities” by Ezeh et al. [13], a new community detection method—able to uncover disjoint clusters of nodes, clusters with overlapping nodes, and single isolated nodes forming a partition with a unique node—was proposed. Differing from previous state-of-the-art methods, the authors proposed an approach which iteratively computes the bridging centrality value of the nodes to find those with the highest bridging centrality value. Once a bridge node has been identified, the algorithm computes the node similarity between the bridge and its neighbors, and the neighbors with the least node similarity values are disconnected. This step is iterated until a stopping criterion condition is satisfied. Simulations on both real-world and synthetic networks demonstrated that the proposed method was able to efficiently classify multi-type nodes in network communities.

In the paper “Predicting the Evolution of Physics Research from a Complex Network Perspective” by Liu et al. [14], the problem of quantitative knowledge evolution in physics research was addressed through complex networks, built on bibliographic coupling and co-citation data extracted from the American Physical Society repository from 1981 to 2010. For each year, the topical clusters (TCs) were uncovered through the Louvain method and compared to subsequent years to assess their similarity. Once this information was gathered, a machine learning model was applied to predict the evolution of the clusters in terms of permanence, disappearance, merging or splitting. This research showed that the number of papers from certain journals, degree, closeness, and betweenness mostly drove the predictor.

In the paper “Uncovering the Dependence of Cascading Failures on Network Topology by Constructing Null Models” by Ding et al. [15], the problem of cascading failures in complex network infrastructures was taken on. The authors analyzed the impact that underlying network topology has on cascading failures in realistic Internet Autonomous System network scenarios by constructing different types of null models. By analyzing the shortest paths in different topological configurations, the results revealed the effects that microscale (e.g., degree distribution, assortativity, and transitivity) and mesoscale (e.g., rich-club and community structure) network properties have on cascade robustness when intentional node attacks are performed.

In the paper “Service-Oriented Model Encapsulation and Selection Method for Complex System Simulation Based on Cloud Architecture” by Xiong et al. [16], a service-oriented model encapsulation and selection method to construct complex system simulation applications was proposed. The method encapsulates models with large computational requirements in shared simulation services in the cloud architecture. It also allows the distributed scheduling of model services and a semantic search framework, useful for the users in searching the required models. An optimization selection algorithm based on quality of service (QoS) was proposed to support users in obtaining an ordered candidate model set satisfying a certain QoS. The performed experiment proved that the proposed method was able to effectively improve the execution efficiency of complex system simulation applications.

In the paper “Minimum Memory-Based Sign Adjustment in Signed Social Networks” by Qi et al. [17], the authors focused on signed social networks—and in particular, on the impact of limited memory on the convergence of the network. The research analyzed random and minimum memory-based sign adjustment rules. Under these rules, the impacts of an initial ratio of positive links, rewiring probability, network size, neighbor number and randomness upon structural balance are compared. The experimental results showed that the minimum memory-based sign adjustment can globally balance the network if the rewiring probability in the Newman–Watts small world model exceeds a critical value. When the rewiring probability is large, the resulting network is denser, and as a consequence, it is easier for the influence of each sign adjustment to spread to the whole network.

In the paper “A SOM-Based Membrane Optimization Algorithm for Community Detection” by Liu et al. [18], an evolutionary membrane community detection algorithm based on self-organizing maps (SOMs) was proposed. Initially, the community detection problem was formulated as a discrete optimization problem. Then, three features typical of the membrane algorithm—objects, reaction rules, and membrane structure—were designed to analyze the characteristics of the community structure. Here, an object was defined as a partition. Genetic algorithms and differential evolution were employed as two reaction rules, to let the objects evolve in different regions of the membrane. Finally, to choose the number of membranes by learning, and to mine the structure of the current objects in the decision space, the SOM was employed. To validate the algorithm, simulations were carried out on both synthetic and real-world networks. The experimental results showed that the proposed algorithm is highly accurate, stable and efficient in the execution when compared to other contestant algorithms.

In the paper “Image Entropy for the Identification of Chimera States of Spatiotemporal Divergence in Complex Coupled Maps of Matrices” by Smidtaite et al. [19], the complex networks of coupled maps of matrices (NCMM) are investigated. The authors proved that an NCMM can achieve two different steady states: quiet or divergence. The analysis of the regions around the boundary lines separating these two steady states showed the existence of chimera states of spatiotemporal divergence. This work demonstrated that for identifying such regions, digital image entropy can be exploited as an effective measure in different networks, including regular, feed-forward, random, and small-world NCMM.

In the paper “Evolution Model of Spatial Interaction Network in Online Social Networking Services” by Dong et al. [20], the research focused on modelling the evolution of spatial interactions between users of online social networks diffusing geospatial information at a city level. Through such interactions, a city interaction network was built. The proposed evolution model of the city interaction network takes into account two dynamics: the edge arrival time and the preferential attachment of the edge. More specifically, six preferential attachment models (Random-Random, Random-Degree, Degree-Random, Geographical distance, Degree-Degree, Degree-Degree-Geographical distance) were considered and compared. The authors found that the degree of the node and the geographic distance of the edge highly influenced the evolution of the spatial interaction network. Moreover, the experiments—comparing the optimal model with the real city interaction network, extracted from the information dissemination of WeChat users—revealed a good matching.

We hope that the selected papers described above will be of interest for the community of physicists, computer scientists and others working in the challenging field of complex networks.
